# Influence of Porosities of 3D Printed Titanium Implants on the Tensile Properties in a Rat Tendon Repair Model

**DOI:** 10.51894/001c.123410

**Published:** 2024-09-09

**Authors:** Michael Fry, Weiping Ren, Therese Bou-Akl, Bin Wu, Paula Pawlitz, David C. Markel

**Affiliations:** 1 Section of Orthopaedic Surgery Ascension Providence Hospital, Southfield, MI, USA; 2 Virotech Co., Inc., Troy, MI, USA; 3 The CORE Institute, Novi, MI, USA

**Keywords:** titanium, porosity, tendon healing

## Abstract

**BACKGROUND:**

There is a desire in orthopaedics to have soft tissue, particularly tendon, grow into metallic implants. With the introduction of three-dimensional (3D) printed porous metal implants, we hypothesized that tendons could directly attach to the implants. However, the effects of the porous metal structure on tissue growth and penetration into the pores are unknown. Using a rat model, we investigated the effect of pore size on tendon repair fixation using 3D printed titanium implants.

**METHODS:**

There were three experimental groups of eight Sprague Dawley rats (n = 24) plus control (n = 3). Implants had defined pore sizes of 400µm (n = 8), 700µm (n = 8), and 1000µm (n = 8). A defect was created in the Achilles tendon and the implant positioned between cut ends and secured with suture. Specimens were harvested at twelve weeks. Half the specimens underwent mechanical testing to assess tensile load to failure. The remaining specimens were fixed and processed for hard tissue histological analysis.

**RESULTS:**

The average load to failure was 72.6N for controls (SD 10.04), 29.95N for 400µm (SD 17.95), 55.08N for 700µm (SD 13.47), and 63.08N for 1000µm (SD 1.87). The load to failure was generally better in the larger pore sizes. The 700µm and 1000µm specimens performed similarly, while the 400µm showed significant differences vs control (p = 0.039), vs 1000µm (p = 0.010), and approached significance vs 700µm (p = 0.066). There was increasing ingrowth as pore size increased. Histology showed fibrous tendon tissue within and around the implants, with collagen fibers organized in bundles.

**CONCLUSIONS:**

Tendon repair utilizing implants with 700µm and 1000µm pores exhibited similar load to failure as controls. Using a defined pore structure at the attachment points of tendons to implants may allow predictable tendon ingrowth onto/into an implant at the time of revision arthroplasty.

## INTRODUCTION

Extensor mechanism and abductor reconstructions in total joint arthroplasty are problematic. Several methods exist for repair, ranging from simple suturing, augmentation with autograft or allograft, to complete replacement of the entire mechanism.^1–3^ The goal of repair is to achieve strong, stable reattachment and restore function. However, outcomes of these procedures are variable, and all treatment options are associated with substantial complications and failure rates.^1–7^

When tendons are injured, they do not fully regain their initial properties. Fibrosis and scar formation occurs, which decreases the mechanical strength of the tendon and can lead to re-injury and chronic complications.^8–10^ In order to improve outcomes after tendon injury, a better understanding of the optimal conditions to promote tendon healing and prevent fibrosis is needed.

The ability to grow tendon directly into a metal implant would have great reconstructive advantages. With the introduction of three dimensional (3D) printed porous metal implants, it was hypothesized that tendons could be grown onto or into the porous implant and thus directly attached. Several studies have demonstrated successful tendon healing and ingrowth of tissue into a porous metal implant,^11–14^ and use of a porous metal implant has been shown to improve tendon healing compared to direct repair.^15–17^ However, the effects of the porous metal structure on tissue growth and penetration into the pores are not fully understood, and there is no consensus regarding optimal pore size and structure to promote tendon healing.

We previously evaluated printed porous titanium (Ti) cylinder constructs with three pore sizes (400µm, 700µm, and 1000µm) on the viability and function of fibroblasts and tenocytes *in-vitro* using a bioreactor. Our results indicated that these cells penetrated and survived inside the material within an optimal pore size of 700µm.^18^

In this study, we sought to build upon the *in-vitro* work to better understand the impact of implant design in terms of tendon healing and strength. Using a rat model, we investigated the effect of pore size on tendon repair fixation using printed Ti implants with defined and differing pore sizes. We hypothesized that tendon repair with larger pore sizes would result in better tissue migration into the implants and thus improved mechanical strength.

## MATERIALS AND METHODS

### Ti Cylinders

**Figure 1.**
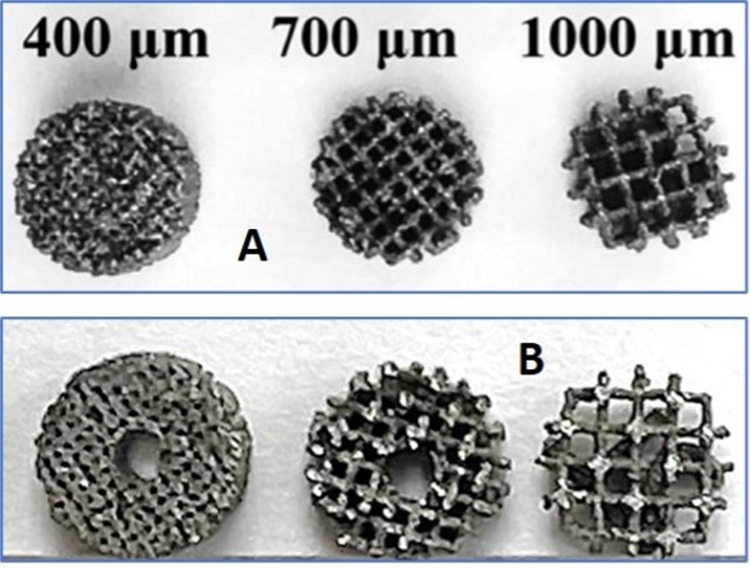
Custom 3D printed porous titanium cylinders (4x2mm). (A) shows the original cylinders and (B) shows the 400μm and 700μm cylinders with the central 1mm hole.

Custom 3D printed porous Ti cylinders 4mm in diameter and 2mm in height with three defined pore sizes (400µm, 700µm, and 1000µm) were provided by Stryker Orthopaedics (Mahwah, NJ). Implants were printed using additive manufacturing technique according to Stryker’s specifications to ensure standardized and defined pore structure. A 1mm diameter hole was drilled through the center of the 400µm and 700µm cylinders to facilitate their fixation between the cut ends of the tendon (Figure 1). Pore sizes were selected based on a range that was manufacturable while also encompassing the ranges used for ingrowth devices.

### Animals and Surgical Technique

**Figure 2.**
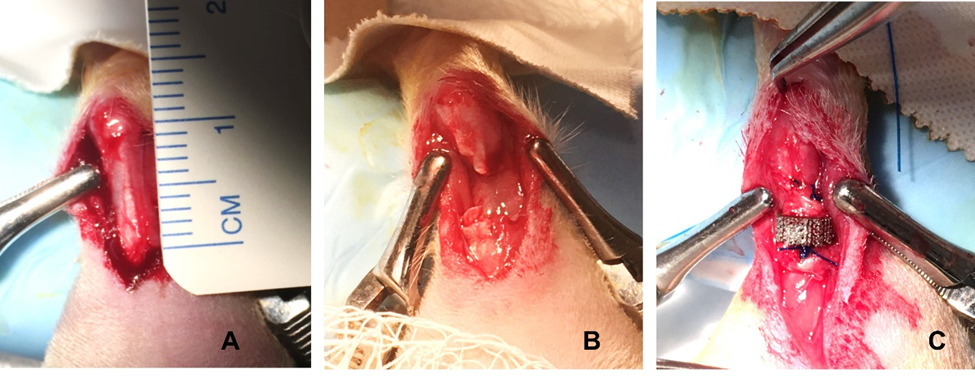
Fixation of Ti implant into Achilles tendon defect using a modified Kessler suture technique.

This animal study was approved by the Institutional Animal Care and Use Committee of Ascension Providence Hospital (Southfield, MI), IACUC # 102-19. Twenty-seven adult male Sprague Dawley rats (body weight of 500-700g) were randomized into three experimental groups based on the implant pore size (n = 8 per group) plus control (n = 3). The rat Achilles tendon injury model has been used frequently to model rupture repair or other tendon pathologies.^19^ The model was established by other investigators to evaluate tendon healing using different suture fixation methods.^20–22^ Animals were anesthetized using isoflurane, and fur was clipped from the right hind leg. The animals were positioned prone, and the surgical site scrubbed with povidone iodine followed by a rinse of alcohol. A 10mm longitudinal incision was made to expose the tendon complex, and an Achilles tendon defect was created by making a transverse cut 3mm above the tendon insertion into the calcaneus. A 2mm full-thickness segment was then removed. The implant was positioned between the two cut ends and sutured in place with 4-0 polypropylene monofilament using a modified Kessler technique (Figure 2). For the control group, a similar 2mm segment was resected and direct repair was performed using the same suture technique. Skin was then closed with a continuous subcuticular 5-0 monocryl suture.

After the surgery, the animals were housed individually, allowed to move freely, and were fed a normal diet. The rats were sacrificed at twelve weeks. The tissue was harvested *en bloc* for analysis, which included the calcaneus, the tendon with implant in place, and the gastrocnemius muscle. Half of the specimens underwent mechanical testing to assess tensile load to failure. The remaining specimens were fixed in 10% neutral buffered formalin (NBF) and processed for hard tissue histological analysis.

### Mechanical Testing

**Figure 3.**
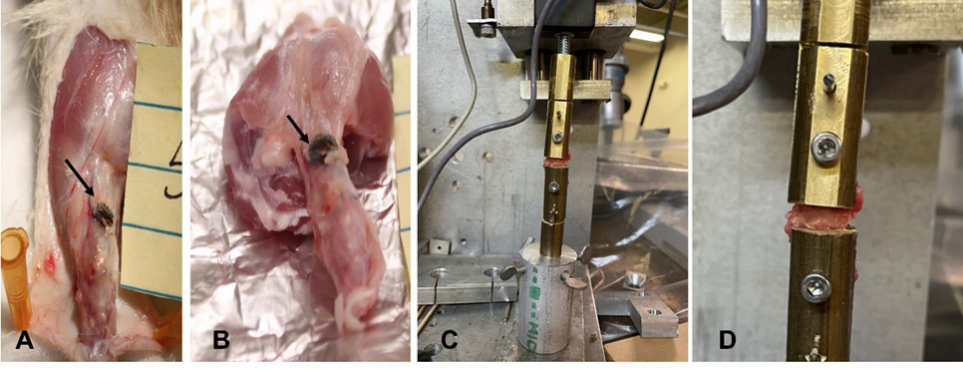
Tensile testing setup. (A and B), the specimen with the implant in place before and after harvesting. Black arrows pointing at the implant (C) the specimen clamped to the tensile testing machine. (D) an enlarged view of the clamped sample.

Tensile load to failure testing was performed at Wayne State University (Detroit, MI) using an electromechanical material testing system and custom fixtures (MTS QTest/100 tensile tester by MTS, MN, USA). Excess soft tissue was trimmed, the calcaneal and gastrocnemial ends of the tendon were coated with cyanoacrylate adhesive to allow for secure fixation. Specimens were fixed onto a tensile testing machine by clamping the gastrocnemius muscle into the upper clamp and the calcaneal end of the tendon to the lower clamp (Figure 3). Care was taken to ensure that the direction of the loading force was in-line with the direction of tendon fibers. Quasi-static tensile loading Testing was performed using a tension to failure of 0.1%/s and stopped once the implant was completely separated from the tendon, and the peak tensile force and the load to failure were recorded.

### Histology

Collected samples with the implant in place were fixed in 10% BNF for 48 hours followed by a rinse in distilled water and then stored in 70% ethanol till processing. Samples processed and embedded in Methyl methacrylate(MMA). Thick longitudinal sections showing the tendon-implant intersection from both ends were obtained (200-300µm) and were stained with hematoxylin and eosin (H&E). All sections were evaluated independently by two pathologists.

### Statistical Analysis

All data were expressed as mean ± standard deviation (SD). The statistical analysis was performed by the first author ( Mike Fry) using the Microsoft Excel data analysis tool pack (Microsoft Office 365, Version 1705), single factor analysis of variance and paired T-tests were used to compare load to failure for all pore sizes. *P* values less than 0.05 were considered significant.

## RESULTS

All animals tolerated the procedure well. No rats were removed from the study due to surgical or animal care complications.

**Figure 4.**
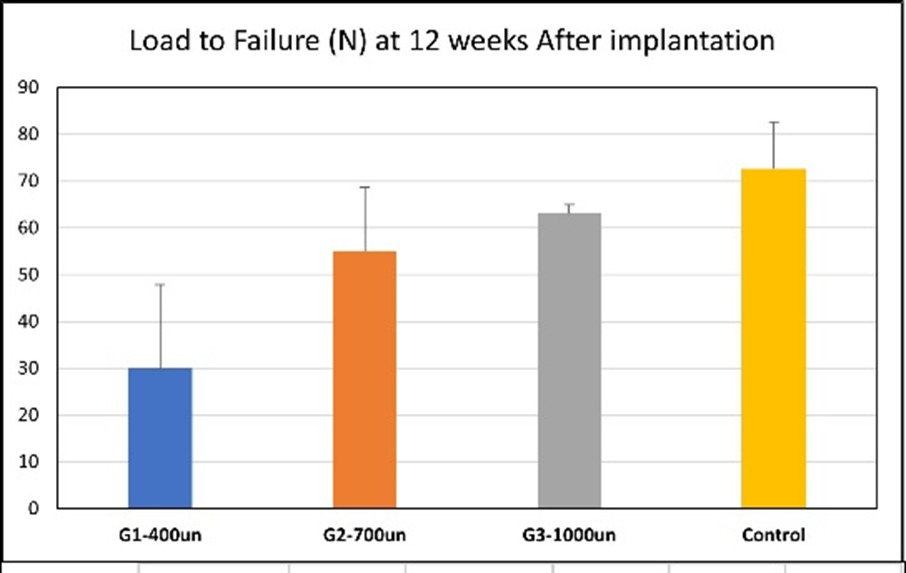
Tensile testing results (load to failure) and statistical analysis.

The tensile testing results are shown in Figure 4, all failures occurred at the site of grafting. The average load to failure was 72.6N for controls (SD 10.04), 29.95N for 400µm (SD 17.95), 55.08N for 700µm (SD 13.47), and 63.08N for 1000µm (SD 1.87). The load to failure was generally better in the larger pore sizes.

No significant differences in the load to failure were noted between control vs 700µm (p = 0.186), control vs 1000µm (p = 0.105), or 700µm vs 1000µm (p = 0.284). The 400µm performed less well and demonstrated significant differences in load to failure vs control (p = 0.039), vs 1000µm (p = 0.010), and approached significance vs 700µm (p = 0.066).

**Figure 5.**
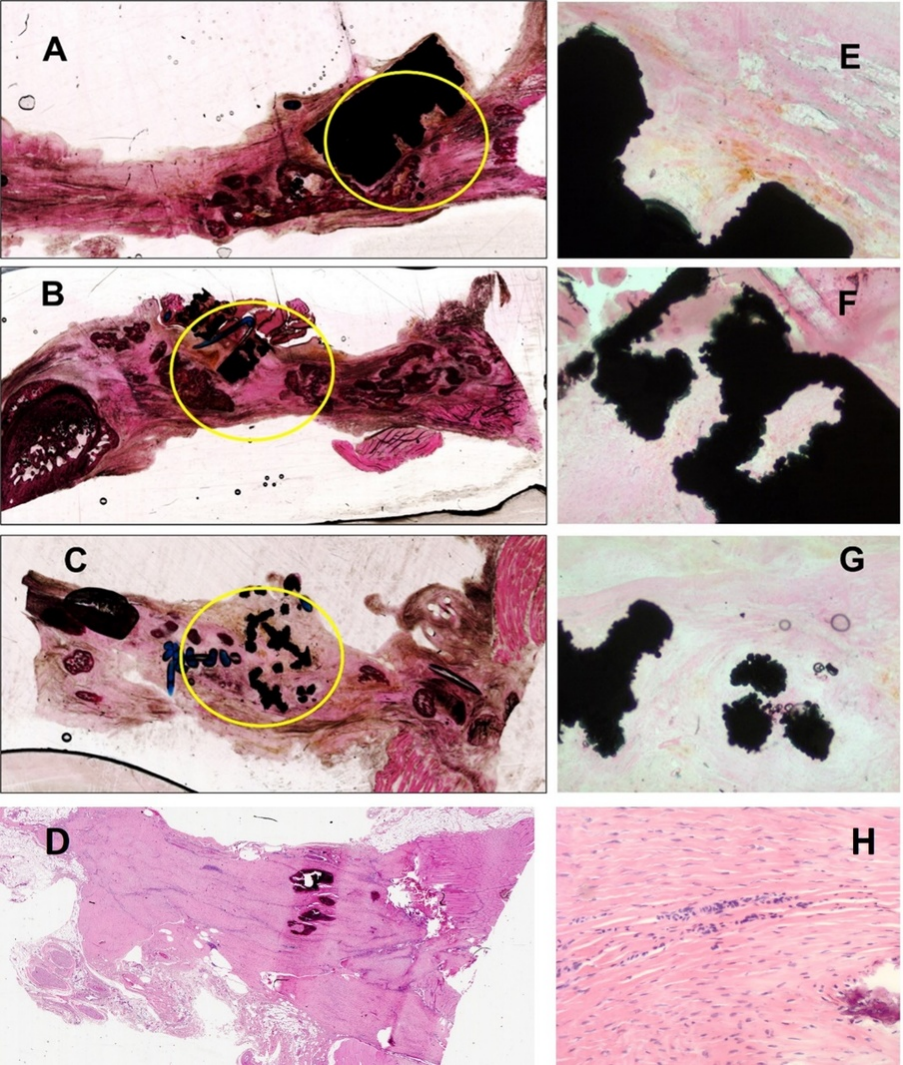
Images of H&E hard tissue sections (200-300µm thick). Left panel show the whole section and the right panel shows higher magnification images of the tissue close to the implant. Yellow circles show the place of the implants within the sections. (A and E) show implants with pore size of 400µm, (B and F) pore size 700µm and (C and G) pore size 1000µm. (D and H) show the whole section and a higher magnification image of the control tendon without implant.

Histological evaluation showed fibrous tendon tissue grown within and around the implant material. The collagen appeared to be organized in bundles (Figure 5). Minimal or no tissue infiltration into the pores of the 400µm samples ( figure 5A and E), with the most infiltration seen within the 1000µm samples ( figure 5C and G). It was not possible technologically to identify the specific cell types and depth of tendon ingrowth into the scaffolds.

## DISCUSSION

The ability to attach tendons directly to metallic implants is needed and desired in orthopaedic surgery. Printed porous titanium implants have the potential to specifically define the pore size in any area, which may allow better promotion of soft-tissue ingrowth into an implant. The structural and biologic mechanisms to achieve this goal remain poorly understood due to the complexity of tendon biology and previously due to the difficulty of creating defined pore structures. In this study, we showed that tendon repair using implants with larger pore sizes (700µm and 1000µm) had improved strength and tissue ingrowth compared to a smaller (400µm) pore size. These findings are consistent with the results of our *in-vitro* study.^18^

Other studies have investigated the effect of pore size on soft tissue healing *in vivo*. Zheng et al used four sizes (657.65µm, 527.15µm, 348.68µm, and 169.20µm) of a porous Ti implant to evaluate supraspinatus tendon healing and strength in rabbits.^23^ They found pore size of 527.15µm to have the greatest tissue ingrowth and tensile strength at both 6 weeks (56.67N) and 12 weeks (90.33N) (Zheng Y). Their histology results also note that implants with pore size of 527.15µm demonstrated the greatest depth of tendon ingrowth (833.33µm), as well as the thickest and most organized muscle fibers, which we were unable to appreciate in our study despite using similar slice thickness in our sections. Guo et al compared Ti implants with three pore sizes (300µm, 500µm, and 700 µm) in a rabbit patellar tendon model, finding pore size of 500µm to have the greatest tissue ingrowth and pull-out strength at 12 weeks.^24^ This study showed that tissue infiltration within the pores increased as the pore diameter increased, and that is in agreement with the findings by Chimutengwende-Gordon et al who implanted porous Ti implants with three pore sizes (500µm, 700µm, and 1000µm) and three strut sizes (200µm, 300µm, and 400µm) into the paraspinal muscles of sheep and evaluated histological soft tissue ingrowth at four weeks.^25^ They noted larger pore sizes exhibited more extensive tissue infiltration and determined that the combination of pore size of 700µm with strut size of 300µm supported revascularization to the greatest degree.^25^

Due to the significant complication rates and failures associated with extensor mechanism and abductor reconstructions,^1–6^ the utility of attaching tendon to implant in total joint arthroplasty is clear. However, soft tissue attachment to a metallic prosthesis has broad use in many areas of orthopaedic surgery. In orthopaedic oncology, custom porous implants have been used during endoprosthetic limb salvage procedures to reattach tendons and ligaments to their insertion points.^26,27^ Also, numerous studies have investigated the use of porous metal implants during rotator cuff repair.^13–16^ Tucker et al found that, compared to standard repair, supraspinatus repair with a porous Ti-coated implant resulted in improved tendon healing and superior mechanical properties in rats.^15^ In a canine model, Higuera et al demonstrated the benefit of allogenic bone plates saturated with recombinant human osteogenic protein-1 (rhOP-1) when reattaching the supraspinatus tendon to a porous Ti anchor.^14^ The potential benefit in sports medicine was further demonstrated in a pig model by Ryu et al, who showed enhancement of tendon-bone interface healing and graft maturation with the use of a cylindrical “titanium-web” during ACL reconstruction.^17^

There were some limitations to the study. First, while we were able to demonstrate statistically significant differences between pore sizes, the size of our study population was relatively small. Second, as previously mentioned we were unable to identify specific cell types or depth of tendon growth into the scaffold. Third, while the implants used were defined in size and shape, they were not “optimized” for any cell type, which has been shown to affect cell affinity and spread.^28^ Optimal pore size and tissue integration into an implant may be different with another structure. However, our results showed an effect of pore size on fixation strength and healing of soft tissues and may provide insight into future research. Additional studies are needed to further define the optimal conditions (structure, growth factors, etc.) that promote tendon healing to an implant. Additional mechanical testing will also be required to define pore sizes that will allow tolerance of clinical conditions.

## CONCLUSION

Printing titanium implants allows for precise determination of pore size and structure. Our results showed that tendon repair utilizing implants with 700µm and 1000µm pores exhibited similar load to failure as controls. Increasing pore size also promoted better ingrowth/ongrowth. Using a defined pore structure at the attachment points of tendons to implants may allow predictable tendon to implant reconstruction at the time of revision arthroplasty.

### Acknowledgments

Stryker Orthopaedics (Mahwah, NJ) kindly provided the custom 3D printed porous Ti cylinders used in this study. The authors would also like to thank Dr. Paul Begeman (Department of Biomedical Engineering, Wayne State University) for his assistance with the mechanical testing.

### Conflict of Interest

The senior author, David Markel, is a paid consultant for Stryker and Smith & Nephew. Dr. Markel serves on the editorial board of the *Journal of Arthroplasty* and *Arthroplasty Today*, and he is a board member for MARCQI and the Michigan Orthopedic Society. The rest of the authors have no conflict of interest to declare.
